# Bringing chiral functionality to in vivo applications of nanomaterials

**DOI:** 10.1038/s41377-022-00841-5

**Published:** 2022-05-27

**Authors:** Maria Mukhina

**Affiliations:** Department of Molecular and Cellular Biology, Harvard University, Cambridge, MA 02138 USA

**Keywords:** Optics and photonics, Optical physics

## Abstract

Chirality is a universal property of an endless number of objects in the universe. Nanotechnology is rapidly expanding to find ways to introduce chirality to artificial nanostructures. In a recent publication in *Light: Science and Applications*, Das et al. have successfully used capping with chiral ligand molecules to obtain chiral carbon dots. The authors provide a theoretical model to describe the origin of chirality in carbon dots as arising due to exciton coupling in a pair of chiral chromophores. Due to non-toxic chemical composition and sizes as small as 2–5 nm, the chiral carbon dots have the potential to outperform other chiral nanostructures in numerous biomedical applications. However, similarly to chiral drugs, their chiral toxicity must be well understood before the carbon dots are brought to living systems.

Chirality is a property of symmetry: a chiral object cannot be superimposed on its mirror image by any translations or rotations, thereby chiral objects always have two forms with left and right handedness. Chiral objects exist at pretty much any scale in the physical world, starting from galaxies down to subatomic particles.

Nanotechnology is making rapidly expanding progress in finding ways to induce chirality in artificial materials with nanoscale features. Collective chirality can be introduced by certain spatial arrangements of non-chiral nanostructures [1]. Individual nanostructures can possess dissymmetry as well, leading to an ensemble with chiral properties. The known origins for individual nanoscale chirality include the natural occurrence of chiral defects such as screw dislocation that propagates through the crystal as a turn of a screw and distorts its lattice [2]; intrinsic chirality of crystal lattice in semiconductor nanocrystals [3]; and chiral shape in metal and semiconductor nanoparticles [4]. An alternative pathway to generate chirality is by placing a single nanoparticle with an achiral core in a chiral environment. Typically, it is done by using chiral ligand molecules during the synthesis [4–6]. In a recent publication in *Light: Science and Applications*, Das et al. have successfully used this strategy to obtain chiral carbon dots [7].

Das et al. utilize Density Functional Theory (DFT) to investigate the origin of chirality in carbon dots obtained through well-established synthetic protocols. They demonstrate that, similarly to organic molecules, chirality in carbon dots arises due to exciton coupling induced by the interaction between a pair of chiral chromophores. The theoretical modeling reveals that peaks with opposite signs observed by Das et al. in the long-wavelength range of circular dichroism spectra can be attributed to the interactions between one or two chiral ligands and the carbon core constituting a dimer of a polycyclic aromatic hydrocarbon (PAH) centers.

Chiral nanostructures can interact selectively with other chiral objects and rotate the plane of polarization of linearly polarized light, giving a rise to optical activity observed in circular dichroism and circularly polarized photoluminescence. These properties open up numerous potential applications, including asymmetric catalysis, enantiomeric separation, chiral sensing, and spintronics [5]. Since most of the biomolecules, including the omnipresent DNA molecule, are chiral, and millions of nanocrystals can fit inside the animal cell, chiral nanostructures have tremendous potential in in vivo applications [8].

In living systems, chiral nanoparticles interact with biological enantiomers through the process called enantioselective recognition, which is driven by the difference in the formation energies of the nanoparticle – biotarget complexes with heterogeneous versus homogeneous stereochemistry (e.g., left-right versus right-right complex, Fig. 1). Enantioselective recognition is involved in most biological applications of the chiral nanomaterials, among which nanozymes for enantioselective biological catalysis and catalytic gene editing represent one of the most promising applications. Carbon dots have the potential to outperform other chiral nanomaterials in enantioselective catalysis due to the combination of low-cost fabrication methods, non-toxic chemical composition, and sizes that can be as small as 2–5 nm [9]. In the recent work [10], cysteine-capped chiral carbon dots have been used successfully as nanozymes mimicking the activity of topoisomerase I.

**Fig. 1:**
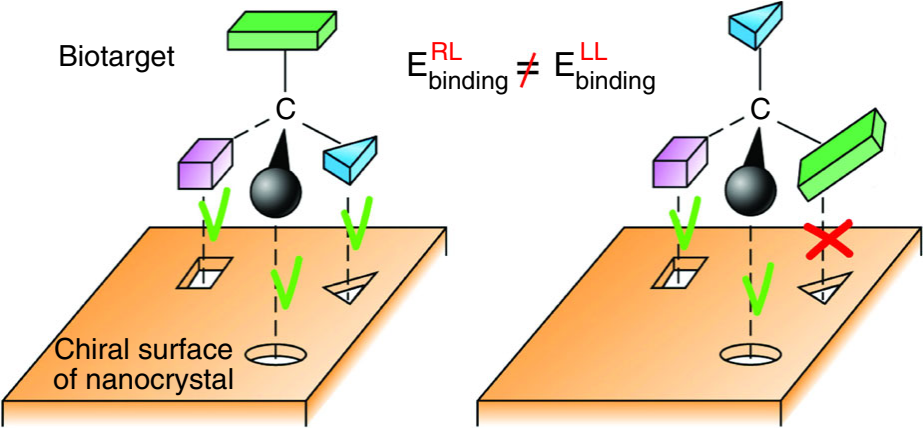
A cartoon showing the process of enantioselective recognition of a chiral biotarget on the chirally distorted surface of a nanocrystal. The process is driven by the difference in the formation energies of the complex with heterogeneous (e.g., right-left) and homogeneous (e.g., left-left) stereochemistry

The process of enantioselective recognition can have very important consequences in the biomedical applications of the chiral nanomaterials, as is true for drugs, many of which are chiral with one enantiomer having a therapeutic effect and another one often being toxic or mutagenic. The thalidomide disaster represents an extreme example of chiral drug toxicity. The left-handed enantiomer of thalidomide causes horrific birth defects, while the right-handed enantiomer has a sedative effect [11]. These considerations lead to the conclusion that the chiral toxicity of the nanocrystals must be well understood before they are brought to biomedical applications. Similar to how it is done with drugs, only enantiopure samples of the nanocrystals can be introduced to biological systems to reduce the risks of adverse effects.
